# Relevance of individual bronchial symptoms for asthma diagnosis and control in patients with rhinitis: A MASK‐air study

**DOI:** 10.1002/clt2.12358

**Published:** 2024-05-28

**Authors:** Bernardo Sousa‐Pinto, Gilles Louis, Rafael J. Vieira, Wienczyslawa Czarlewski, Josep M. Anto, Rita Amaral, Ana Sá‐Sousa, Luisa Brussino, G. Walter Canonica, Claudia Chaves Loureiro, Alvaro A. Cruz, Bilun Gemicioglu, Tari Haahtela, Maciej Kupczyk, Violeta Kvedariene, Desirée E. Larenas‐Linnemann, Nhân Pham‐Thi, Francesca Puggioni, Frederico S. Regateiro, Jan Romantowski, Joaquin Sastre, Nicola Scichilone, Luis Taborda‐Barata, Maria Teresa Ventura, Ioana Agache, Anna Bedbrook, Alida Benfante, Karl C. Bergmann, Sinthia Bosnic‐Anticevich, Matteo Bonini, Louis‐Philippe Boulet, Guy Brusselle, Roland Buhl, Lorenzo Cecchi, Denis Charpin, Elisio M. Costa, Stefano Del Giacco, Marek Jutel, Ludger Klimek, Piotr Kuna, Daniel Laune, Mika Makela, Mario Morais‐Almeida, Rachel Nadif, Marek Niedoszytko, Nikolaos G. Papadopoulos, Alberto Papi, Oliver Pfaar, Daniela Rivero‐Yeverino, Nicolas Roche, Boleslaw Samolinski, Mohamed H. Shamji, Aziz Sheikh, Charlotte Suppli Ulrik, Omar S. Usmani, Arunas Valiulis, Arzu Yorgancioglu, Torsten Zuberbier, Joao A. Fonseca, Benoit Pétré, Renaud Louis, Jean Bousquet, MASK‐air think tank

**Affiliations:** ^1^ MEDCIDS ‐ Department of Community Medicine, Information and Health Decision Sciences Faculty of Medicine University of Porto Porto Portugal; ^2^ Faculty of Medicine CINTESIS@RISE ‐ Health Research Network University of Porto Porto Portugal; ^3^ Department of Public Health University of Liège Liège Belgium; ^4^ GIGA I3 Research Group University of Liège Liège Belgium; ^5^ Medical Consulting Czarlewski Levallois France; ^6^ MASK‐air Montpellier France; ^7^ ISGlobal Barcelona Institute for Global Health Barcelona Spain; ^8^ Universitat Pompeu Fabra (UPF) Barcelona Spain; ^9^ CIBER Epidemiología y Salud Pública (CIBERESP) Barcelona Spain; ^10^ Department of Medical Sciences University of Torino Torino Italy; ^11^ Allergy and Clinical Immunology Unit Mauriziano Hospital Torino Italy; ^12^ Department of Biomedical Sciences Humanitas University Milan Italy; ^13^ Asthma and Allergy Unit IRCCS Humanitas Research Hospital Milan Italy; ^14^ Department of Pneumology Faculty of Medicine Coimbra University Hospital Coimbra Portugal; ^15^ Coimbra Institute for Clinical and Biomedical Research, CIBB Coimbra Portugal; ^16^ Fundaçao ProAR Federal University of Bahia and GARD/WHO Planning Group Salvador Bahia Brazil; ^17^ Department of Pulmonary Diseases Faculty of Medicine Istanbul University‐Cerrahpaşa Cerrahpaşa Istanbul Turkey; ^18^ Institute of Pulmonology and Tuberculosis Istanbul University‐Cerrahpaşa Istanbul Turkey; ^19^ Skin and Allergy Hospital Helsinki University Hospital and University of Helsinki Helsinki Finland; ^20^ Division of Internal Medicine, Asthma and Allergy Barlicki University Hospital Medical University of Lodz Lodz Poland; ^21^ Institute of Clinical Medicine Clinic of Chest Diseases and Allergology Faculty of Medicine Vilnius University Vilnius Lithuania; ^22^ Department of Pathology Institute of Biomedical Sciences Faculty of Medicine Vilnius University Vilnius Lithuania; ^23^ Center of Excellence in Asthma and Allergy Médica Sur Clinical Foundation and Hospital México City Mexico; ^24^ Ecole Polytechnique de Palaiseau Palaiseau France; ^25^ IRBA (Institut de Recherche Bio‐Médicale des Armées) Brétigny sur Orge France; ^26^ Université Paris Cité Paris France; ^27^ IRCCS Humanitas Research Center Personalized Medicine Asthma & Allergy Rozzano, Milan Italy; ^28^ Allergy and Clinical Immunology Unit Centro Hospitalar e Universitário de Coimbra Coimbra Portugal; ^29^ Faculty of Medicine Center for Innovative Biomedicine and Biotechnology (CIBB) University of Coimbra Coimbra Portugal; ^30^ Faculty of Medicine Institute of Immunology University of Coimbra Coimbra Portugal; ^31^ UBIAir ‐ Clinical & Experimental Lung Centre and CICS‐UBI Health Sciences Research Centre University of Beira Interior Covilhã Portugal; ^32^ Department of Allergology Medical University of Gdańsk Gdansk Poland; ^33^ Allergy Service Fundacion Jimenez Diaz Autonoma University of Madrid CIBERES‐ISCIII Madrid Spain; ^34^ PROMISE Department University of Palermo Palermo Italy; ^35^ Department of Immunoallergology Cova da Beira University Hospital Centre Covilhã Portugal; ^36^ Allergy and Clinical Immunology University of Bari Medical School Bari Italy; ^37^ Institute of Sciences of Food Production National Research Council (ISPA‐CNR) Bari Italy; ^38^ Faculty of Medicine Transylvania University Brasov Brasov Romania; ^39^ ARIA Montpellier France; ^40^ Institute of Allergology Charité—Universitätsmedizin Berlin Corporate Member of Freie Universität Berlin and Humboldt‐Universität zu Berlin Berlin Germany; ^41^ Fraunhofer Institute for Translational Medicine and Pharmacology ITMP Immunology and Allergology Berlin Germany; ^42^ Quality Use of Respiratory Medicines Group Woolcock Institute of Medical Research Sydney New South Wales Australia; ^43^ Macquarie Medical School Macquarie University Macquarie Park New South Wales Australia; ^44^ Department of Public Health and Infectious Diseases Sapienza University of Rome Rome Italy; ^45^ National Heart and Lung Institute (NHLI) Imperial College London London UK; ^46^ Quebec Heart and Lung Institute Laval University Quebec City Quebec Canada; ^47^ Department of Respiratory Medicine Ghent University Hospital Ghent Belgium; ^48^ Department of Pulmonary Medicine Mainz University Hospital Mainz Germany; ^49^ SOS Allergology and Clinical Immunology USL Toscana Centro Prato Italy; ^50^ Clinique des Bronches Allergie et Sommeil Hôpital Nord Marseille France; ^51^ CINTESIS@RISE Biochemistry Lab Faculty of Pharmacy and Competence Center on Active and Healthy Ageing University of Porto Porto Portugal; ^52^ Department of Medical Sciences and Public Health and Unit of Allergy and Clinical Immunology University Hospital “Duilio Casula” University of Cagliari Cagliari Italy; ^53^ Department of Clinical Immunology Wrocław Medical University Wroclaw Poland; ^54^ ALL‐MED Medical Research Institute Wroclaw Poland; ^55^ Department of Otolaryngology, Head and Neck Surgery Universitätsmedizin Mainz Mainz Germany; ^56^ Center for Rhinology and Allergology Wiesbaden Germany; ^57^ KYomed INNOV Montpellier France; ^58^ Allergy Center CUF Descobertas Hospital Lisbon Portugal; ^59^ Université Paris‐Saclay UVSQ University Paris‐Sud Villejuif France; ^60^ Inserm Equipe d’Epidémiologie Respiratoire Intégrative CESP Villejuif France; ^61^ Allergy Department 2nd Pediatric Clinic University of Athens Athens Greece; ^62^ Respiratory Medicine Department of Translational Medicine University of Ferrara Ferrara Italy; ^63^ Section of Rhinology and Allergy Department of Otorhinolaryngology Head and Neck Surgery University Hospital Marburg Philipps‐Universität Marburg Marburg Germany; ^64^ Servicio de Alergia e Inmunología clínica Hospital Universitario de Puebla Puebla México; ^65^ Pneumologie AP‐HP Centre Université de Paris Cité Hôpital Cochin Paris France; ^66^ UMR 1016 Institut Cochin Paris France; ^67^ Department of Prevention of Environmental Hazards Allergology and Immunology Medical University of Warsaw Warsaw Poland; ^68^ NIHR Imperial Biomedical Research Centre London UK; ^69^ Usher Institute The University of Edinburgh Edinburgh UK; ^70^ Department of Respiratory Medicine Copenhagen University Hospital‐Hvidovre Copenhagen Denmark; ^71^ Institute of Clinical Medicine University of Copenhagen Copenhagen Denmark; ^72^ Airways Disease Section Royal Brompton Hospital London UK; ^73^ Interdisciplinary Research Group of Human Ecology Institute of Clinical Medicine and Institute of Health Sciences Medical Faculty of Vilnius University Vilnius Lithuania; ^74^ European Academy of Paediatrics (EAP/UEMS‐SP) Brussel Belgium; ^75^ Department of Pulmonary Diseases Faculty of Medicine Celal Bayar University Manisa Turkey; ^76^ Department of Pulmonary Medicine CHU Liège Liège Belgium; ^77^ MASK‐air think tank—See Section 6

**Keywords:** asthma, diagnosis, dyspnea, mHealth, wheezing

## Abstract

**Rationale:**

It is unclear how each individual asthma symptom is associated with asthma diagnosis or control.

**Objectives:**

To assess the performance of individual asthma symptoms in the identification of patients with asthma and their association with asthma control.

**Methods:**

In this cross‐sectional study, we assessed real‐world data using the MASK‐air^®^ app. We compared the frequency of occurrence of five asthma symptoms (dyspnea, wheezing, chest tightness, fatigue and night symptoms, as assessed by the Control of Allergic Rhinitis and Asthma Test [CARAT] questionnaire) in patients with probable, possible or no current asthma. We calculated the sensitivity, specificity and predictive values of each symptom, and assessed the association between each symptom and asthma control (measured using the e‐DASTHMA score). Results were validated in a sample of patients with a physician‐established diagnosis of asthma.

**Measurement and Main Results:**

We included 951 patients (2153 CARAT assessments), with 468 having probable asthma, 166 possible asthma and 317 no evidence of asthma. Wheezing displayed the highest specificity (90.5%) and positive predictive value (90.8%). In patients with probable asthma, dyspnea and chest tightness were more strongly associated with asthma control than other symptoms. Dyspnea was the symptom with the highest sensitivity (76.1%) and the one consistently associated with the control of asthma as assessed by e‐DASTHMA. Consistent results were observed when assessing patients with a physician‐made diagnosis of asthma.

**Conclusions:**

Wheezing and chest tightness were the asthma symptoms with the highest specificity for asthma diagnosis, while dyspnea displayed the highest sensitivity and strongest association with asthma control.

## 1 INTRODUCTION

Asthma guidelines such as the Global INitiative for Asthma (GINA) have improved the knowledge and management of asthma.^1^ However, (i) there is a substantial number of asthma misdiagnoses (under/over‐diagnosis) in primary care, partly associated with the under‐use of spirometers and under‐reporting of symptoms by patients,^2^ (ii) many patients diagnosed with asthma are insufficiently monitored and (iii) poor adherence to treatment remains frequent.^3^ These aspects are all associated with poor control, increased risk of asthma attacks and increased healthcare utilization.^4^, ^5^

The aspects related to asthma treatment adherence, follow‐up, and control may be partly addressed by patient‐centered digital solutions allowing the collection of real‐world data on asthma symptoms and treatment. The EU Commission has proposed digital solutions, such as wearables and mHealth apps, to engage citizens in the self‐management of chronic diseases.^6^ Thus, the patient perspective, captured by patient‐reported outcome measures (PROMs), can strengthen patient‐centered care.^7^ Although various mHealth apps have been developed to support asthma monitoring and management in recent years^8^, ^9^, very few have been developed for both asthma diagnosis and asthma control using validated PROMs. One of the apps, MASK‐air^®^ (Mobile Airways Sentinel networK), is a Good Practice of Directorate‐General Health and Food Safety^10^ and the only one listed as a Best Practice of Organisation for Economic Cooperation and Development for chronic diseases.^10^ It includes several validated asthma PROMs.^11^, ^12^, ^13^ One such PROM is the Control of Allergic Rhinitis and Asthma Test (CARAT) questionnaire, aiming to assess asthma and AR control together. It has 10 questions addressing upper and lower airway symptoms, sleep disturbances, limitation of activities and the need to increase medication over the previous four weeks.^14^, ^15^, ^16^ CARAT includes three questions on key asthma symptoms (wheezing, dyspnea and chest tightness).

mHealth tools and other patient‐centered digital solutions can help address the misdiagnosis of asthma not only through the assessment of directly provided daily patient data but also by the use of such data to identify specific variables associated with asthma diagnosis or control. In particular, mHealth‐based direct patient data may allow us to assess whether some specific asthma symptoms or symptom patterns are associated with asthma diagnosis or control. This is in line with the recent European Respiratory Society guidelines for the diagnosis of asthma in adults, which, in their conclusion, highlighted that more research should be undertaken on the value of symptoms to predict accurate asthma diagnoses.^17^

In this study, we used the MASK‐air^®^ PROMs data (CARAT) to assess how individual asthma symptoms (wheezing, dyspnea, chest tightness) were associated with both asthma diagnosis and control. Knowing which symptom is the most associated with asthma can help reduce the important number of misdiagnoses in the clinical practice, while knowing which symptom has the greatest impact on asthma control is fundamental in the guidance of strategies aiming to improve daily patient management.

## 2 METHODS

### 2.1 Study design

A full description of the Methods is available in the Online Data Supplement. In this cross‐sectional study, we used MASK‐air^®^ data to compare the frequency of occurrence (over the previous month) of five asthma symptoms (dyspnea, wheezing, chest tightness, fatigue and night symptoms) in patients with probable asthma, possible asthma and no evidence of asthma^18^ as assessed by CARAT. Results of this study were validated in a sample of patients in whom asthma diagnosis had been assessed by a physician in the context of a transfer of innovation project (Twinning) of the European Innovation Partnership on Active and Healthy Ageing.

### 2.2 Settings and participants

MASK‐air^®^ is freely available in 27 countries. In this study, we included data from MASK‐air^®^ users from May 21, 2015 to December 2021, reporting MASK‐air^®^ data in at least three different months. The users (i) had a self‐reported diagnosis of allergic rhinitis and (ii) ranged in age from 16 to 90 years (or lower than 16 years in countries with a lower age of digital consent).^19^, ^20^ We also included data from participants of the Twinning project who were enrolled during a medical consultation with an asthma specialist.^18^ Asthma was diagnosed according to GINA,^1^ with patients having a pulmonary function test and answering the CARAT questionnaire. Following that consultation, participants were classified as having “current asthma” or “no evidence of (current or past) asthma”.

### 2.3 Ethics

An Independent Review Board (Bohn‐Köln) approval was obtained for the MASK‐air studies.^21^ For the Twinning project, additional local review board approvals were obtained, and written consent was provided by the patients. All data were anonymized before the study, and users agreed to the analysis of their data.

### 2.4 Data sources and variables

The MASK‐air^®^ app comprises a daily monitoring questionnaire assessing (i) the impact of asthma and rhinitis symptoms on a daily basis by means of 0–100 visual analog scales (VASs) (with a higher score corresponding to a higher impact of allergy symptoms) and (ii) the daily use of asthma and rhinitis medication (available from country‐specific lists with prescribed and over‐the‐counter medications).^19^ The symptom and medication information provided in the MASK‐air^®^ daily monitoring questionnaire allows for the computation of the e‐DASTHMA, a 0–100 score assessing the daily control of asthma.^22^

In addition to the daily monitoring questionnaire, MASK‐air^®^ also includes (although in a non‐daily basis) CARAT, a questionnaire assessing the control of allergic rhinitis and asthma in the previous 4 weeks (Table E1).^15^

### 2.5 Data analysis

When responding to the MASK‐air^®^ daily monitoring questionnaire, it is not possible to skip any of the questions, and data are saved to the dataset only after the final answer. This precludes any missing data. All analyses were performed using software R (version 4.0.0).

Using a two‐step approach, k‐means cluster analysis methods were applied to group MASK‐air^®^ users on their probability of having asthma. Obtained clusters subsequently enabled the classification of patients as having “probable asthma”, “possible asthma” or “no evidence of asthma” (i.e., rhinitis alone).^18^

For patients with “probable asthma”, “possible asthma” or “no evidence of asthma”, using the CARAT questions,^12^, ^15^, ^16^ we assessed the frequency of having at least one day per week of (i) dyspnea, (ii) wheezing, (iii) chest tightness, (iv) tiredness/limitations in doing tasks and (v) night symptoms. For each question, we calculated its sensitivity, specificity and predictive values. We also assessed the performance of each question in the discrimination between “possible asthma” and “probable asthma”. As each patient could have answered CARAT more than once, we considered, in our main analysis, the first CARAT reported by each patient. We then performed a sensitivity analysis considering (i) all reported CARAT questionnaires or (ii) pre‐COVID‐19 data only. To validate the obtained results, the sensitivity, specificity and predictive values of each symptom were assessed in a sample of patients in whom the diagnosis of asthma was established by a physician (Twinning participants).

Finally, in patients with probable asthma, we computed the median e‐DASTHMA by each category of each CARAT question to assess the most discriminative symptom regarding asthma control. We considered both the median and maximal e‐DASTHMA levels for the 4 weeks before answers to CARAT were provided.

## 3 RESULTS

### 3.1 Demographic characteristics

**FIGURE 1:**
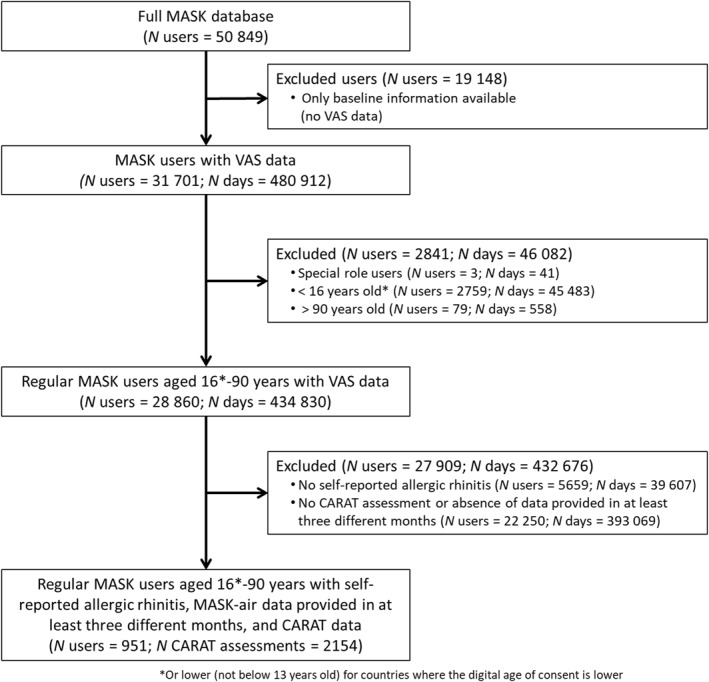
Flow chart illustrating participants' selection.

We included 951 patients with rhinitis (62.8% women; mean ± SD age = 38.6 ± 13.6), of which 468 (49.2%) had probable asthma, 166 (17.5%) possible asthma and 317 (33.3%) no evidence of asthma. The demographic characteristics of the patients are presented in Tables 1 and 2 and Figure 1). There were 2154 CARAT assessments (Table 3 and Table E2), with 302 indicating controlled CARAT and 1852 indicating uncontrolled complete CARAT. For all these participants, e‐DASTHMA information was available.

**TABLE 1:** Demographic characteristics of the patients.

	Main analysis (*N* = 951)
Females—*N* (%)	597 (62.8)
Age—mean (SD)	38.6 (13.6)
Self‐reported asthma—*N* (%)	393 (41.3)
Asthma classification—*N* (%)	
Probable asthma	468 (49.2)
Possible asthma	166 (17.5)
No evidence of asthma	317 (33.3)
Self‐reported conjunctivitis—*N* (%)	625 (65.7)

	Twinning sample (*N* = 283)
Females—*N* (%)	162 (57.2)
Age—mean (SD)	43.4 (17.2)
Asthma classification diagnosis—*N* (%)	
Current asthma	97 (34.3)
No evidence of asthma	186 (65.7)
Conjunctivitis—*N* (%)	148 (52.3)

Abbreviation: SD, Standard‐deviation.

**TABLE 2:** Frequency of MASK‐air^®^ users per country.

Country	*N* (%)
Argentina	19 (2.0)
Australia	6 (0.6)
Austria	17 (1.8)
Belgium	9 (0.9)
Brazil	19 (2.0)
Canada	3 (0.3)
Czech Republic	6 (0.6)
Denmark	3 (0.3)
Finland	16 (1.7)
France	102 (10.7)
Germany	94 (9.9)
Great Britain	14 (1.5)
Greece	36 (3.8)
Hungary	12 (1.3)
Italy	65 (6.8)
Japan	29 (3.0)
Lebanon	4 (0.4)
Lithuania	113 (11.9)
Mexico	82 (8.6)
Netherlands	20 (2.1)
Poland	62 (6.5)
Portugal	77 (8.1)
Slovenia	14 (1.5)
Spain	59 (6.2)
Sweden	11 (1.2)
Switzerland	13 (1.4)
Turkey	46 (4.8)

**TABLE 3:** Number of CARAT questionnaires provided by user.

Number of CARAT questionnaires answered by patient	*N* patients (%)
1	694 (73.0)
2	154 (16.2)
3	32 (3.4)
4	20 (2.1)
5	13 (1.4)
6–10	22 (2.3)
>10	16 (1.7)

Abbreviation: CARAT, control of allergic rhinitis and asthma test.

### 3.2 Association between individual asthma symptoms and asthma diagnosis

We compared patients with “no evidence of asthma” versus those with “probable or possible asthma” on the presence of each asthma symptom as assessed by CARAT. We examined the first CARAT questionnaire reported by each patient (*N* = 951, Table 4; Figure 1) and observed that the specificity decreased from wheezing (90.5%) to chest tightness, dyspnea, night symptoms and fatigue (51.1%). On the other hand, the sensitivity was around 50% for wheezing and chest tightness and increased up to 63% for dyspnea. The positive predictive value (PPV) ranged from 71.6% to 90.8% (patients with wheezing had a 90.8% risk of having asthma) and the negative predictive value (NPV) was always lower than 50%.

**TABLE 4:** Comparison between patients classified as having “possible or probable asthma” versus “no evidence of asthma” on the ever occurrence of asthma symptoms as assessed by the Control of Allergic Rhinitis and Asthma Test (CARAT).

CARAT questions	Sensitivity—% (95% CI)	Specificity—% (95% CI)	PPV—% (95% CI)	NPV—% (95% CI)
A. First CARAT assessment by user				
Shortness of breath/dyspnea^a^	65.5 (61.8–69.2)	68.5 (63.3–73.6)	80.6 (77.2–84.0)	49.8 (45.1–54.5)
Wheezing in the chest	46.8 (43.0–50.7)	90.5 (87.3–93.8)	90.8 (87.7–94.0)	46.0 (42.1–49.9)
Chest tightness	48.1 (44.2–52.0)	84.5 (80.6–88.5)	86.2 (82.6–89.8)	44.9 (40.9–48.9)
Tiredness/limitations doing tasks	65.1 (61.4–68.9)	51.1 (45.6–56.7)	72.7 (69.0–76.4)	42.3 (37.3–47.2)
Night symptoms	59.1 (55.3–63.0)	53.0 (47.5–58.5)	71.6 (67.7–75.4)	39.3 (34.7–44.0)
B. All CARAT assessments				
Shortness of breath/dyspnea^b^	75.2 (73.1–77.2)	69.6 (65.2–73.8)	90.4 (88.9–91.9)	42.2 (38.7–45.8)
Wheezing in the chest	49.9 (47.5–52.2)	90.6 (87.9–93.3)	95.3 (93.9–96.7)	32.1 (29.5–34.6)
Chest tightness	58.4 (56.1–60.7)	84.5 (81.2–87.9)	93.5 (92.1–95.0)	34.7 (31.9–37.5)
Tiredness/limitations doing tasks	71.1 (69.0–73.3)	52.2 (47.6–56.9)	85.1 (83.2–86.9)	32.1 (28.7–35.5)
Night symptoms	57.2 (54.9–59.6)	49.8 (45.1–54.4)	81.3 (79.1–83.6)	23.3 (20.6–26.1)

Abbreviations: NPV, negative predictive value; PPV, positive predictive value.

^a^ Considering data before 2020 only: Sensitivity = 59.5%; Specificity = 66.7%; PPV = 83.1%; NPV = 37.4%.

^b^ Considering data before 2020 only: Sensitivity = 64.8%; Specificity = 68.9%; PPV = 89.5%; NPV = 32.6%.

Similar results were observed when all CARAT assessments were reported (Table 4) or when considering pre‐COVID‐19 data only (Table 4).

**FIGURE 2:**
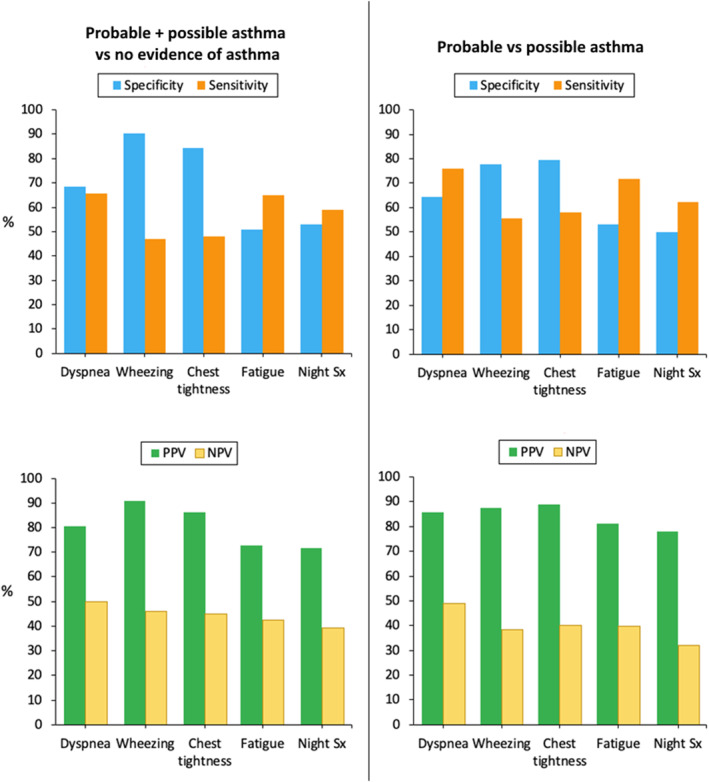
Sensitivity, specificity, positive predictive value and negative predictive value of the occurrence of specific asthma symptoms (Sx; as assessed by the control of allergic rhinitis and asthma test) on asthma diagnosis.

In a subsequent analysis, we compared patients with “probable asthma” versus those with “possible asthma” on the presence of each asthma symptom as assessed by CARAT. When considering only the first CARAT questionnaires reported by the patients (Figure 2; Table 5), the specificity decreased from chest tightness (79.5%) and wheezing (77.7%) to dyspnea (64.5%) and fatigue and night symptoms (50.0%). On the other hand, the sensitivity was around 55% for wheezing and chest tightness and increased up to 76.1% for dyspnea. The PPV ranged from 77.9% to 88.9%. Furthermore, the NPV was always lower than 50%. Similar results were observed when all CARAT assessments were reported (Table 5) or when considering pre‐COVID‐19 data only (Table 5).

**TABLE 5:** Comparison between patients classified as having “probable asthma” versus “possible asthma” on the ever occurrence of asthma symptoms, as assessed by the control of allergic rhinitis and asthma test.

CARAT questions	Sensitivity—% (95% CI)	Specificity—% (95% CI)	PPV—% (95% CI)	NPV—% (95% CI)
A. First CARAT assessment by user				
Shortness of breath/dyspnea^a^	76.1 (72.2–79.9)	64.5 (57.2–71.7)	85.8 (82.4–89.1)	48.9 (42.2–55.5)
Wheezing in the chest	55.6 (51.1–60.1)	77.7 (71.4–84.0)	87.5 (83.8–91.3)	38.3 (33.1–43.5)
Chest tightness	57.9 (53.4–62.4)	79.5 (73.4–85.7)	88.9 (85.3–92.4)	40.1 (34.8–45.4)
Tiredness/limitations doing tasks	71.6 (67.5–75.7)	53.0 (45.4–60.6)	81.1 (77.3–84.9)	39.8 (33.7–46.3)
Night symptoms	62.4 (58.0–66.8)	50.0 (42.4–57.6)	77.9 (73.7–82.1)	32.0 (26.4–37.7)
B. All CARAT assessments				
Shortness of breath/dyspnea^b^	80.9 (78.9–82.9)	57.6 (51.6–63.7)	91.6 (90.1–93.1)	34.7 (30.1–39.2)
Wheezing in the chest	54.0 (51.4–56.6)	73.7 (68.3–79.1)	92.1 (90.3–93.9)	22.0 (19.2–24.7)
Chest tightness	63.2 (60.7–65.7)	69.0 (63.3–74.7)	92.1 (90.4–93.8)	24.8 (21.6–28.0)
Tiredness/limitations doing tasks	76.4 (74.2–78.7)	58.8 (52.8–64.9)	91.4 (89.8–92.9)	30.4 (26.4–34.5)
Night symptoms	59.4 (56.9–62.0)	55.3 (49.1–61.4)	88.3 (86.3–90.3)	19.3 (16.5–22.2)

Abbreviations: NPV, negative predictive value; PPV, positive predictive value.

^a^ Considering data before 2020 only: Sensitivity = 69.3%; Specificity = 70.8%; PPV = 87.8%; NPV = 43.2%.

^b^ Considering data before 2020 only: Sensitivity = 71.0%; Specificity = 67.6%; PPV = 92.0%; NPV = 30.9%.

**FIGURE 3:**
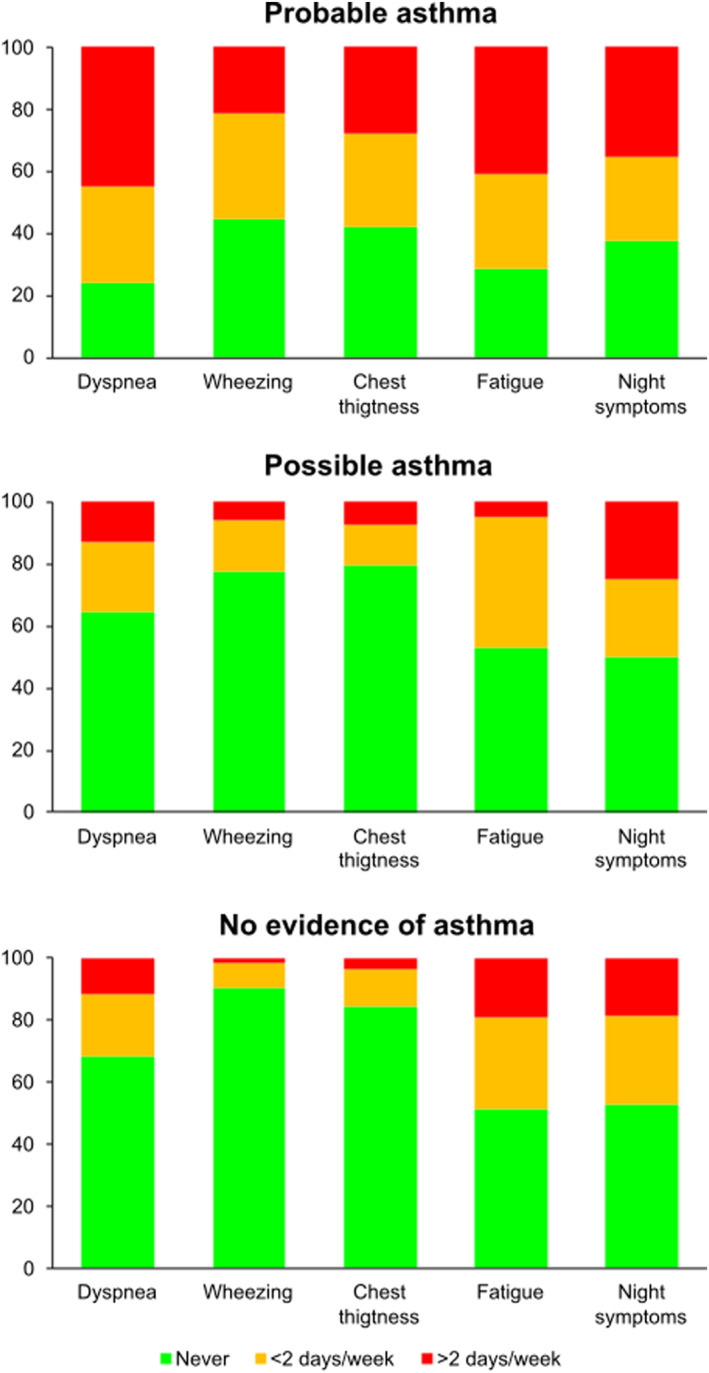
Frequency of symptoms reported by the control of allergic rhinitis and asthma test in patients with probable asthma.

When considering the first CARAT questionnaire reported by each patient, only 21% of patients reported having never experienced dyspnea in the previous 4 weeks versus 28% for fatigue, 38% for night symptoms, 42% for chest tightness and 44% for wheezing (Figure 3). On the other hand, 45% of patients reported an average of more than 2 days per week of dyspnea, 41% reported the same for fatigue, 35% for night symptoms, 28% for chest tightness and 21% for wheezing.

### 3.3 Validation of results in a sample of patients assessed by a physician

We analyzed 283 Twinning participants, of whom 97 (34.3%) had current asthma and 186 had no evidence of asthma, as assessed by a physician (Table 1). As observed in our main analysis, wheezing was the symptom which displayed the highest specificity (86.0%) and PPV (70.5%), while dyspnea was the symptom associated with the highest sensitivity (76.3%) and NPV (85.6%) (Table 6). The sensitivity and specificity for each symptom were similar to those observed in the main analysis. On the other hand, a lower PPV and a higher NPV were observed in the Twinning study, reflecting differences in the proportion of patients with asthma.

**TABLE 6:** Comparison between participants of the Twinning project classified as having “current asthma” versus “no evidence of asthma” on the ever occurrence of asthma symptoms, as assessed by the control of allergic rhinitis and asthma test.

CARAT questions	Sensitivity—% (95% CI)	Specificity—% (95% CI)	PPV—% (95% CI)	NPV—% (95% CI)
Shortness of breath/dyspnea	76.3 (67.8–84.8)	73.7 (67.3–80.0)	60.2 (51.5–68.8)	85.6 (80.2–91.1)
Wheezing in the chest	63.9 (54.4–73.5)	86.0 (81.0–91.0)	70.5 (60.9–80.0)	82.1 (76.7–87.4)
Chest tightness	55.7 (45.8–65.6)	80.1 (74.4–85.8)	59.3 (49.2–69.4)	77.6 (71.7–83.5)
Tiredness/limitations doing tasks	64.9 (55.5–74.4)	65.1 (58.2–71.9)	49.2 (40.6–57.9)	78.1 (71.5–84.6)
Night symptoms	47.4 (37.5–57.4)	68.8 (62.2–75.5)	44.2 (34.7–53.8)	71.5 (64.9–78.1)

Abbreviations: NPV, negative predictive value; PPV, positive predictive value.

### 3.4 Association between individual asthma symptoms and asthma control

Dyspnea was the symptom the most strongly associated with e‐DASTHMA levels (Table 7). In fact, for dyspnea, the median e‐DASTHMA ranged from 5.7 (IQR = 9.5] in patients who had never reported that symptom in the past 4 weeks to 27.8 (IQR = 30.0) in those who reported the symptom every day or almost every day (high effect size = 0.97). Chest tightness and wheezing had a heterogeneous impact on e‐DASTHMA (high to no effect). Fatigue and night symptoms had a more consistent effect, with medium effect sizes being observed when comparing median e‐DASTHMA levels for patients who had experienced those symptoms every day (or almost) versus those who had never experienced them in the previous 4 weeks. Only dyspnea had a consistent effect on e‐DASTHMA levels when all CARAT questionnaires were considered (Table 7).

**TABLE 7:** Median e‐DASTHMA in patients with probable asthma according to the frequency of specific asthma symptoms as assessed by the control of allergic rhinitis and asthma test.

CARAT questions	Never—median (IQR) [*N*]	Up to 2 days per week—median (IQR) [*N*]	More than 2 days per week—median (IQR) [*N*]	Everyday or almost—median (IQR) [*N*]	Effect size			
					Never versus < 2 days/week	<2 days versus +2 days/week	+2 days/week versus every day or almost	Never versus any day/week
A. First CARAT assessment by user (*N* = 468)								
Shortness of breath/dyspnea	5.7 (9.5) [112]	12.3 (18.8) [145]	20.9 (24.6) [107]	27.8 (30.0) [104]	0.64	0.56	0.34	0.97
Wheezing in the chest	10.2 (18.0) [208]	15.2 (20.5) [161]	27.1 (34.3) [55]	29.1 (23.7) [44]	0.37	0.55	0.08	0.53
Chest tightness	8.3 (18.0) [197]	14.7 (18.0) [141]	31.0 (28.9) [60]	26.5 (26.9) [70]	0.55	0.85	−0.20	0.77
Tiredness/task limitations	8.1 (11.8) [133]	13.7 (22.4) [144]	19.6 (24.3) [93]	24.5 (26.8) [98]	0.45	0.37	0.27	0.74
Night symptoms	9.1 (17.8) [176]	13.4 (19.7) [126]	20.1 (29.4) [94]	24.4 (25.6) [72]	0.36	0.37	0.21	0.63
B. All CARAT assessments (*N* = 1452)								
Shortness of breath/dyspnea	6.8 (11.3) [277]	12.3 (14.9) [436]	31.2 (31.4) [350]	35.6 (21.7) [389]	0.57	1.07	0.22	0.93
Wheezing in the chest	13.3 (19.1) [668]	18.8 (31.7) [488]	39.8 (32.0) [190]	38.1 (26.3) [106]	0.34	1.20	−0.10	0.65
Chest tightness	13.5 (21.5) [534]	15.0 (17.7) [370]	38.3 (29.6) [296]	33.5 (36.2) [252]	0.11	1.61	−0.22	0.45
Tiredness/task limitations	10.1 (12.3) [343]	12.7 (18.5) [510]	33.4 (26.1) [267]	33.4 (23.4) [332]	0.24	1.25	0	0.73
Night symptoms	17.8 (36.5) [589]	20.6 (23.0) [324]	22.5 (29.5) [153]	13.2 (25.0) [386]	0.14	0.10	−0.56	0.03

*Note*: IQR, interquartile range; Effect size: small: 0.20–0.49, medium: 0.50–0.79, high ≥0.80.

## 4 DISCUSSION

This is the first study to assess individual asthma symptoms for the diagnosis and control of the disease. Although all symptoms were associated with asthma diagnosis, wheezing was the symptom with the highest specificity and PPV for diagnosis. Dyspnea was the most sensitive symptom associated with the control of asthma as assessed by a novel daily electronic symptom‐medication score (e‐DASTHMA).

### 4.1 Strengths and limitations

This study has several limitations. First, the main analysis was not performed in patients with a physician‐made diagnosis of asthma. However, we did not solely rely on self‐reported asthma (as asthma‐self‐reporting tends to be associated with the overestimation or the underestimation of asthma) but rather classified patients as having “probable asthma”, “possible asthma” and “no evidence of asthma” based on self‐reported asthma status, use of asthma medication and daily asthma symptoms. In a previous study assessing a sample of patients with physician‐confirmed diagnosis, we observed that patients clustered as having “probable asthma” had a physician diagnosis of current or past asthma in 92.3% of cases, while patients clustered as having “no evidence of asthma” had a physician diagnosis of “no current asthma” in 90.4% of cases.^18^ In addition, in this study, we assessed a sample of patients in whom the diagnosis of asthma was established by a physician (Twinning), obtaining consistent results with those of our main analysis. Of note, we did not solely assess patients from asthma clinics with a confirmed diagnosis of asthma since we wanted to have as many patients as possible and in a real‐life context.

All assessed patients displayed self‐reported rhinitis. Therefore, results are valid only for patients with nasal symptoms, who do however represent a very large proportion of patients with asthma.^23^ We could not test patients with asthma and without rhinitis as they represent less than 10% of patients with asthma in the MASK‐air^®^ database.

Some of the symptoms examined (e.g. dyspnea, wheezing, chest tightness, and fatigue) are rather nonspecific and may be the expression of different diseases (e.g. asthma and chronic obstructive pulmonary disease (COPD)), and also of the response to treatment in terms of control.^24^

Additionally, we were not able to assess all asthma symptoms. For example, we did not have information on the presence of cough and secretions. Nevertheless, secretions appear to be less common than wheezing, dyspnea, chest tightness and sleep disturbances. Importantly, treatments received prior to or during the time when the patients used the MASK‐air app may have influenced the relationship between diagnosis and symptoms.

The tools used have been validated. CARAT is a validated questionnaire of asthma.^16^ CARAT displays (i) an internal consistency similar to that of the Asthma Control Test (ACT), (ii) a reliability similar to that of the Asthma Control Questionnaire (ACQ) and higher than that of ACT, (iii) a correlation with clinical assessment of asthma control higher than that of ACT (despite being lower than that of ACQ) and (iv) an AUC‐ROC for asthma higher than that of the ACQ.^16^ e‐DASTHMA is a daily data‐driven asthma control medication score based on MASK‐air^®^ data. It is strongly correlated with daily dyspnea symptoms and moderately correlated with work‐ and quality‐of‐life‐related comparators. It had high test‐retest reliability and displayed moderate‐to‐high responsiveness. e‐DASTHMA was validated in an external cohort of asthma patients enrolled by physicians^25^ and was associated with the GINA classification of asthma control.^1^

We performed the analysis on the first CARAT questionnaires reported and used all CARAT questionnaires as a sensitivity analysis.

### 4.2 Interpretation of the results

The first important message is the significance of wheezing in the diagnosis of asthma. Although PPV is influenced by disease prevalence, the high PPV of wheezing for asthma diagnosis is consistent with a study in a secondary care center with a precise asthma diagnosis.^26^ However, wheezing lacks sensitivity and 45% of patients with asthma did not report this symptom. Chest tightness displayed a high PPV and a high specificity. It was the symptom showing the highest specificity in differentiating probable from possible asthma. By contrast, dyspnea was the symptom with the highest sensitivity. Despite this result, sensitivity was close to 80%, indicating that approximately one fifth of asthma patients do not experience dyspnea, which likely reflects the good control of the disease in that subset of patients. According to the SpIN and SnOUT rules,^27^ a test with high specificity, when positive, helps to rule in the disease. This is the case of wheezing and chest tightness. On the other hand, a test with high sensitivity, when negative, helps to rule out the disease. This may be the case for dyspnea.

The second important message is the significance of dyspnea in asthma control assessment. Dyspnea is a multidimensional, subjective perception of breathing difficulty, commonly seen in patients affected by respiratory diseases, among others.^28^ Although dyspnea perception may differ between subjects^29^ (decreasing with the worsening of asthma, in older adults or in patients with depression^30^), the present study suggests that it is a cardinal symptom associated with asthma control. In a previous MASK‐air^®^ study, VAS asthma was correlated with VAS dyspnea in patients with different degrees of severity, with a particularly strong correlation being observed in severe asthmatic patients.^31^ Dyspnea was the symptom which mostly affected the asthma‐related quality of life in both mild^26^ and severe asthma patients. In a large cross‐sectional study encompassing the whole disease severity spectrum, asthma‐related quality of life was essentially determined by the level of asthma control.^32^ These results were also confirmed in a longitudinal study.^33^ Fatigue and night symptoms were much less specific, and their relationship with asthma control appeared less solid than that of dyspnea and chest tightness.

Future studies may assess whether individual respiratory symptoms can also be useful in the distinction between asthma and other respiratory diseases (namely, COPD).

### 4.3 Generalizability

This study was conducted with patients from 27 countries. Previous MASK‐air^®^ studies have pointed to a high similarity of outcomes in different countries.^34^

## 5 CONCLUSION

Among MASK‐air^®^ users who have rhinitis, wheezing and chest tightness are the symptoms that best predict the presence of asthma (displaying high specificity and PPV), while dyspnea is the symptom that showed the highest sensitivity and the strongest relationship with the level of asthma control. These results highlight the importance of thoroughly assessing individual symptoms during both the initial and follow‐up assessments of patients with suspected or confirmed asthma.

## THE MASK‐AIR THINK TANK

The members of the MASK‐air think tank are to be acknowledged for having enrolled patients in the study (i.e., for suggesting to their patients to install and use the MASK‐air® app). These members include: Marcus Maurer, (1, 2) Rute Almeida, (3, 4) Marek Kulus, (5) Ignacio J Ansotegui, (6) Fulvio Braido, (7, 8) Victoria Cardona, (9) Carlos Robalo Cordeiro, (10) Cemal Cingi, (11) Wytske J Fokkens, (12) Govert de Vries, (13) Antonio FM Giuliano, (14) Tomohisa Linuma, (15) Juan Carlos Ivancevich, (16) Cristina Jácome, (3, 4) Igor Kaidashev, (17) Helga Kraxner, (18) Olga Lourenço, (19) Michael Makris, (20) Ralph Mösges, (21, 22) Joaquim Mullol, (23, 24) Marine Savouré, (25, 26) Robyn O’Hehir, (27) Yoshitaka Okamoto, (28, 29) Markus Ollert, (30‐32) Heidi Olze, (33) Vincenzo Patella, (34‐36) Philip W Rouadi, (37, 38) Sietze Reitsma, (12) Monica Rodriguez‐Gonzalez, (39) Faradiba S Serpa, (40) Mikhail Sofiev, (41) Milan Sova, (42) Annette Sperl, (43) Ana Todo‐Bom, (44) Peter V Tomazic, (45) Ioanna Tsiligianni, (46) Erkka Valovirta, (47) Michiel van Eerd, (13) Mihaela Zidarn, (48, 49) Hubert Blain, (50) Thomas Casale, (51) Tomas Chivato, (52) Jaime Correia‐de‐Sousa, (53) Christopher Corrigan, (54) Frédéric de Blay, (55, 56) Philippe Devillier, (57) Mark Dykewicz, (58) Alessandro Fiocchi, (59) Mattia Giovannini, (60, 61) Ewa Jassem, (62) Thomas Keil, (63) Stefania La Grutta, (64) Brian Lipworth, (64) Jean‐Louis Pépin, (65, 66) Santiago Quirce, (67) Maria J Torres, (68) Sanna Toppila‐Salmi, (69)

1. Institute of Allergology, Charité – Universitätsmedizin Berlin, Corporate Member of Freie Universität Berlin and Humboldt‐Universität zu Berlin, Berlin, Germany.
2. Fraunhofer Institute for Translational Medicine and Pharmacology ITMP, Allergology and Immunology, Berlin, Germany.
3. MEDCIDS ‐ Department of Community Medicine, Information and Health Decision Sciences; Faculty of Medicine, University of Porto, Porto, Portugal.
4. CINTESIS@RISE– Health Research Network, Faculty of Medicine, University of Porto, Porto, Portugal.
5. Department Of Pediatric Respiratory Diseases and Allergology, Medical University of Warsaw, Poland.
6. Department of Allergy and Immunology, Hospital Quironsalud Bizkaia, Bilbao, Spain.
7. Respiratory Clinic, Department of Internal Medicine, University of Genoa, Genoa, Italy
8. IRCCS Ospedale Policlinico San Martino, Genoa, Italy.
9. Allergy Section, Department of Internal Medicine, Hospital Vall d'Hebron & ARADyAL research network, Barcelona, Spain.
10. Centre of Pneumology, Coimbra University Hospital, Portugal.
11. Eskisehir Osmangazi University, Medical Faculty, ENT Department, Eskisehir,Turkey.
12. Department of Otorhinolaryngology, Amsterdam University Medical Centres, Amsterdam, the Netherlands.
13. Peercode BV, Geldermalsen,The Netherlands.
14. Allergy and Clinical Immunology, University of Bari Medical School, Bari, Italy.
15. Ciba University, Chiba, Japan.
16. Servicio de Alergia e Immunologia, Clinica Santa Isabel, Buenos Aires, Argentina.
17. Poltava State Medical University, Ukraine.
18. Department of Otorhinolaryngology, Head and Neck Surgery, Semmelweis University, Budapest, Hungary.
19. Faculty of Health Sciences and CICS – UBI, Health Sciences Research Centre, University of Beira Interior, Covilhã, Portugal.
20. Allergy Unit "D Kalogeromitros", 2nd Dpt of Dermatology and Venereology, National & Kapodistrian University of Athens, "Attikon" University Hospital, Greece.
21. Institute of Medical Statistics and Computational Biology, University of Cologne, Cologne, Germany.
22. ClinCompetence Cologne GmbH, Cologne, Germany.
23. Rhinology Unit & Smell Clinic, ENT Department, Hospital Clínic, Barcelona, Spain.
24. Clinical & Experimental Respiratory Immunoallergy, IDIBAPS, CIBERES, University of Barcelona, Barcelona, Spain.
25. Université Paris‐Saclay, UVSQ, Univ. Paris‐Sud, Inserm, Equipe d’Epidémiologie Respiratoire Intégrative, CESP, Villejuif, France.
26. INSERM U1018, Centre de Recherche en Epidémiologie et Santé des Populations (CESP) / INSERM U1018, Center for Epidemiology and Population Health (CESP)
27. Department of Allergy, Immunology and Respiratory Medicine, Central Clinical School, Monash University and Alfred Health, Melbourne, Victoria, Australia
28. Chiba Rosai Hospital, Chiba, Japan.
29. Chiba University Hospital, Chiba, Japan.
30. Department of Infection and Immunity, Luxembourg Institute of Health, Esch‐sur‐Alzette, Luxembourg
31. Department of Dermatology and Allergy Centre, Odense University Hospital, Odense, Denmark.
32. Odense Research Center for Anaphylaxis « ORCA », Odense, Denmark.
33. Department of Otorhinolaryngology, Head and Neck Surgery Charité ‐ Universitätsmedizin Berlin, corporate member of Freie Universität Berlin, Humboldt‐Universität zu Berlin, and Berlin Institute of Health Berlin Germany.
34. Division of Allergy and Clinical Immunology, Department of Medicine, "Santa Maria della Speranza" Hospital, Battipaglia, Salerno, Italy.
35. Agency of Health ASL, Salerno, Italy.
36. Postgraduate Programme in Allergy and Clinical Immunology, University of Naples Federico II, Naples, Italy.
37. Department of Otolaryngology‐Head and Neck Surgery, Eye and Ear University Hospital, Beirut, Lebanon.
38. Department of Otorhinolaryngology‐Head and Neck Surgery, Dar Al Shifa Hospital, Salmiya, Kuwait.
39. Pediatric Allergy and Clinical Immunology, Hospital Espanol de Mexico, Mexico City Mexico.
40. Asthma Reference Center ‐ School of Medicine of Santa Casa de Misericórdia of Vitória, Espírito Santo, Brazil.
41. Finnish Meteorological Institute (FMI), Helsinki, Finland.
42. Department of Respiratory Medicine and Tuberculosis, University Hospital, Brno, Czech Republic.
43. Department of Otolaryngology, Head and Neck Surgery, Universitätsmedizin Mainz, Mainz, and Center for Rhinology and Allergology, Wiesbaden, Germany.
44. Imunoalergologia, Centro Hospitalar Universitário de Coimbra and Faculty of Medicine, University of Coimbra, Portugal.
45. Dept of General ORL, H&NS, Medical University of Graz, ENT‐University Hospital Graz, Austria.
46. Health Planning Unit, Department of Social Medicine, Faculty of Medicine, University of Crete, Greece and International Primary Care Respiratory Group IPCRG, Aberdeen, Scotland.
47. Department of Lung Diseases and Clinical Immunology, University of Turku and Terveystalo allergy clinic, Turku, Finland.
48. University Clinic of Respiratory and Allergic Diseases, Golnik, Slovenia.
49. University of Ljubljana, Faculty of Medicine, Ljubljana, Slovenia.
50. Department of Geriatrics, Montpellier University hospital, MUSE, Montpellier, France.
51. Division of Allergy/Immunology, University of South Florida, Tampa, FLA, USA.
52. School of Medicine, University CEU San Pablo, Madrid, Spain.
53. Life and Health Sciences Research Institute (ICVS), School of Medicine, University of Minho, Braga, PT Government Associate Laboratory, Braga/Guimarães, Portugal.
54. Division of Asthma, Allergy & Lung Biology, MRC & Asthma UK Centre in Allergic Mechanisms of Asthma, King's College London, London, UK.
55. Allergy Division, Chest Disease Department, University Hospital of Strasbourg, Strasbourg, France.
56. Federation of Translational Medicine, University of Strasbourg, Strasbourg, France.
57. VIM Surrenes, UMR 0892, Pôle des Maladies des Voies Respiratoires, Hôpital Foch, Université Paris‐Saclay, Suresnes, France.
58. Section of Allergy and Immunology, Saint Louis University School of Medicine, Saint Louis, Missouri, USA.
59. Division of Allergy, Department of Pediatric Medicine ‐ The Bambino Gesù Children's Research Hospital Holy see, IRCCS, Rome, Italy.
60. Department of Health Sciences, University of Florence, Florence, Italy.
61. Allergy Unit, Meyer Children's Hospital IRCCS, Florence, Italy.
62. Medical University of Gdańsk, Department of Pneumology, Gdansk, Poland.
63. Institute of Social Medicine, Epidemiology and Health Economics, Charité ‐ Universitätsmedizin Berlin, Berlin; Institute for Clinical Epidemiology and Biometry, University of Wuerzburg, Berlin and Institute of Health Resort Medicine and Health Promotion, Bavarian Health and Food Safety Authority, Bad Kissingen, Germany
64. Institute of Translational Pharmacology, National Research Council, Palermo, Italy.
65. Université Grenoble Alpes, Laboratoire HP2, Grenoble, France.
66. INSERM, U1042 and CHU de Grenoble, France.
67. Department of Allergy, Hospital La Paz Institute for Health Research (IdiPAZ), Madrid, Spain.
68. Allergy Unit, Málaga Regional University Hospital‐IBIMA, Málaga, Spain.
69. Skin and Allergy Hospital, Helsinki University Hospital, and University of Helsinki, Helsinki, Finland.

## AUTHOR CONTRIBUTIONS

Bernardo Sousa‐Pinto and Gilles Louis participated in the study design, data analysis, and manuscript writing (original draft). Jean Bousquet, Benoit Pétré and Renaud Louis participated in the conceptualisation, study design, data analysis, supervision, and manuscript writing (original draft). Torsten Zuberbier, Josep M Anto, and Joao A Fonseca participated in the study design, supervision, and manuscript writing (revision and editing). All other authors participated in data collection and manuscript writing (revision and editing).

## CONFLICT OF INTEREST STATEMENT

JB reports personal fees from Cipla, Menarini, Mylan, Novartis, Purina, Sanofi‐Aventis, Teva, Uriach, other from KYomed‐Innov, and other from Mask‐air‐SAS, outside the submitted work. OP reports grants and personal fees from ALK‐Abelló, grants and personal fees from Allergopharma, grants and personal fees from Stallergenes Greer, grants and personal fees from HAL Allergy Holding B.V./HAL Allergie GmbH, grants from Bencard Allergie GmbH/Allergy Therapeutics, grants from Lofarma, grants and personal fees from ASIT Biotech Tools S.A., grants and personal fees from Laboratorios LETI/LETI Pharma, grants and personal fees from GlaxoSmithKline, personal fees from ROXALL Medizin, personal fees from Novartis, grants and personal fees from Sanofi‐Aventis and Sanofi‐Genzyme, personal fees from Med Update Europe GmbH, personal fees from streamedup! GmbH, grants from Pohl‐Boskamp, grants from Inmunotek S.L., personal fees from John Wiley and Sons, AS, personal fees from Paul‐Martini‐Stiftung (PMS), personal fees from Regeneron Pharmaceuticals Inc., personal fees from RG Aerztefortbildung, personal fees from Institut für Disease Management, personal fees from Springer GmbH, grants and personal fees from AstraZeneca, personal fees from IQVIA Commercial, personal fees from Ingress Health, personal fees from Wort&Bild Verlag, personal fees from Verlag ME, personal fees from Procter&Gamble, personal fees from ALTAMIRA, personal fees from Meinhardt Congress GmbH, personal fees from Deutsche Forschungsgemeinschaft, personal fees from Thieme, grants from Deutsche AllergieLiga e.V., personal fees from AeDA, personal fees from Alfried‐Krupp Krankenhaus, personal fees from Red Maple Trials Inc., outside the submitted work. LPB reports grants from Amgen, AstraZeneca, GlaxoSmithKline, Merck, Novartis, Sanofi‐Regeneron, personal fees from Astra Zeneca, Novartis, GlaxoSmithKline, Merck, Sanofi‐Regeneron, personal fees from AstraZeneca, Covis, GlaxoSmithKline, Novartis, Merck, Sanofi, outside the submitted work. VK reports other from NORAMEDA, outside the submitted work. SDG reports grants from AstraZeneca, grants from GSK, grants from Novartis, grants from Sanofi, outside the submitted work. RL reports and Grants from GSK, Chiesi and AZ and adboard and lecture fees from AZ, GSK, Chiesi. PK reports personal fees from Adamed, personal fees from Berlin Chemie Menarini, personal fees from Boehringer Ingelheim, personal fees from AstraZeneca, personal fees from Glenmark, personal fees from Novartis, personal fees from Polpharma, personal fees from GSK, personal fees from Sanofi, personal fees from Chiesi, personal fees from Celon Pharma, outside the submitted work. TZ reports grants and personal fees from Novartis, grants and personal fees from Henkel, personal fees from Bayer, personal fees from FAES, personal fees from Astra Zeneca, personal fees from AbbVie, personal fees from ALK, personal fees from Almirall, personal fees from Astellas, personal fees from Bayer, personal fees from Bencard, personal fees from Berlin Chemie, personal fees from FAES, personal fees from Hal, personal fees from Leti, personal fees from Mesa, personal fees from Menarini, personal fees from Merck, personal fees from MSD, personal fees from Novartis, personal fees from Pfizer, personal fees from Sanofi, personal fees from Stallergenes, personal fees from Takeda, personal fees from Teva, personal fees from UCB, personal fees from Henkel, personal fees from Kryolan, personal fees from L'Oreal, outside the submitted work; and Organizational affiliations: Commitee member: WHO‐Initiative "Allergic Rhinitis and Its Impact on Asthma" (ARIA); Member of the Board: German Society for Allergy and Clinical Immunology (DGAKI); Head: European Centre for Allergy Research Foundation (ECARF); President: Global Allergy and Asthma European Network (GA2LEN); Member: Committee on Allergy Diagnosis and Molecular Allergology, World Allergy Organization (WAO). MJ reports personal fees from ALK‐Abello, personal fees from Allergopharma, personal fees from Stallergenes, personal fees from Anergis, personal fees from Allergy Therapeutics, personal fees from Leti, and personal fees from HAL, during the conduct of the study; personal fees from GSK, personal fees from Novartis, personal fees from Teva, personal fees from Takeda, and personal fees from Chiesi, outside the submitted work. AC reports personal fees from AstraZeneca, personal fees from Chiesi, personal fees from GSK, personal fees from SANOFI, personal fees from Novartis, personal fees from Boehringer Ingelheim, personal fees from Glennmark, personal fees from Eurofarma, personal fees from Abdi Ibrahim, and personal fees from CROSSJECT, outside the submitted work. LTB reports personal fees from AstraZeneca and personal fees from Diater, outside the submitted work. MK reports personal fees from Astra Zeneca, personal fees from GSK, personal fees from Adamed, personal fees from Polpharma, personal fees from Celon Pharma, personal fees from Berlin Chemie Menarini, personal fees from Novartis, personal fees from Nexter Allergopharma, personal fees from Teva, personal fees from Chiesi, personal fees from Zentiva, personal fees from Sanofi Aventis, personal fees from Pfizer, personal fees from Abbvie, personal fees from Lekam, outside the submitted work. LC reports personal fees from Thermofisher, personal fees from Sanofi, personal fees from Astra Zeneca, personal fees from Novartis, outside the submitted work. NP reports personal fees from NOVARTIS, personal fees from NUTRICIA, personal fees from HAL, personal fees from MENARINI/FAES FARMA, personal fees from SANOFI/REGENERON, personal fees from MYLAN/MEDA, personal fees from BIOMAY, personal fees from AstraZeneca, personal fees from GSK, personal fees from MSD, personal fees from ASIT BIOTECH, personal fees from Boehringer Ingelheim, grants from CAPRICARE, grants from Gerolymatos Int, grants from NUTRICIA, personal fees from MEDCARE, personal fees from ALK, personal fees from OM PHARMA, from BBOTT, outside the submitted work. RB reports grants from Boehringer Ingelheim, GlaxoSmithKline, Novartis and Roche, personal fees from AstraZeneca, Berlin‐Chemie, Boehringer Ingelheim, Chiesi, Cipla, GlaxoSmithKline, Novartis, Sanofi, Roche and Teva, outside the submitted work. JF reports and being co‐founder of an SME that develops mHealth technologies, such as digital biomarkers and has the copyright of the CARAT anda CARATkids PROM. FR reports personal fees from Novartis, personal fees from Sanofi, personal fees from AstraZeneca, personal fees from GSK, personal fees from Medinfar, personal fees from Azentis, outside the submitted work. JS reports grants and personal fees from SANOFI, personal fees from GSK, personal fees from NOVARTIS, personal fees from ASTRA ZENECA, personal fees from MUNDIPHARMA, and personal fees from FAES FARMA, outside the submitted work. DLL reports personal fees from ALK, Astrazeneca national and global, Bayer, Chiesi, Grunenthal, Grin, GSK national and global, Viatris, Menarini, MSD, Novartis, Pfizer, Sanofi, Siegfried, UCB, Carnot, grants from Abbvie, Bayer, Lilly, Sanofi, Astrazeneca, Lilly, Pfizer, Novartis, Circassia, UCB, GSK, Purina institute, outside the submitted work. TH reports personal fees from Orion Pharma, outside the submitted work. AP reports grants from CHIESI, ASTRAZENECA, GSK, SANOFI, personal fees from CHIESI, ASTRAZENECA, GSK, NOVARTIS, SANOFI, IQVIA, AVILLION, ELPEN PHARMACEUTICALS, personal fees from CHIESI, ASTRAZENECA, GSK, BI, MENARINI, NOVARTIS, ZAMBON, MUNDIPHARMA, TEVA, SANOFI, EDMOND PHARMA, IQVIA, MSD, AVILLION, ELPEN PHARMACEUTICALS, outside the submitted work. NR reports grants and personal fees from Boehringer Ingelheim, grants and personal fees from Novartis, grants and personal fees from GSK, personal fees from AstraZeneca, personal fees from Chiesi, grants and personal fees from Pfizer, personal fees from Sanofi, personal fees from Zambon, personal fees from MSD, personal fees from Austral, outside the submitted work. CSU reports grants and personal fees from AZ, grants and personal fees from GSK, personal fees from Chiesi, personal fees from Orion Pharma, grants and personal fees from Sanofi, personal fees from TEVA, personal fees from Pfizer, grants and personal fees from BI, and personal fees from Novartis, outside the submitted work. SBA reports grants from TEVA, personal fees from TEVA, personal fees from TEVA, personal fees from AstraZeneca, personal fees from AstraZeneca, personal fees from Boehringer Ingelheim, personal fees from Boehringer Ingelheim, personal fees from GSK, personal fees from Sanofi, personal fees from Mylan, and personal fees from Menarini, outside the submitted work. The other authors have no COIs to disclose outside the submitted work.

## Supporting information

Supporting Information S1

## Data Availability

Individual participant data underlying the results reported in this Article can be made available (after de‐identification) between 12 and 36 months after Article publication. These data can be supplied to researchers who provide a methodologically sound proposal. Proposals should be directed to the corresponding author (jean.bousquet@orange.fr). We made every effort to follow the EU General Data Protection Regulation; therefore, we can transfer data only if there is a protocol and an agreement between the owner of the data and the person (or institution) requesting the data. To gain access, data requestors will need to sign a data access agreement.
