# Supplementary material for: Relevance of individual bronchial symptoms for asthma diagnosis and control in patients with rhinitis: A MASK‐air study
Source: Clin Transl Allergy. 2024 May 28;14(6):e12358. doi: 10.1002/clt2.12358 (PMC11304469; doi:10.1002/clt2.12358)
Supplement: Supplementary file 1 — Supporting Information S1 [file CLT2-14-e12358-s001.docx]

**Online Data Supplement**

**Relevance of individual bronchial symptoms for asthma diagnosis and control in patients with rhinitis: A MASK-air study**

Bernardo Sousa-Pinto, Gilles Louis, Rafael J Vieira, Wienczyslawa Czarlewski, Josep M Anto, Rita Amaral, Ana Sá-Sousa, Luisa Brussino, G Walter Canonica, Claudia Chaves Loureiro, Alvaro A Cruz, Bilun Gemicioglu, Tari Haahtela, Maciej Kupczyk, Violeta Kvedariene, Desirée E Larenas-Linnemann, Nhân Pham-Thi, Francesca Puggioni, Frederico S Regateiro, Jan Romantowski, Joaquin Sastre, Nicola Scichilone, Luis Taborda-Barata, Maria Teresa Ventura, Ioana Agache, Anna Bedbrook, Elida Benfante, Karl C Bergmann, Sinthia Bosnic-Anticevich, Matteo Bonini, Louis-Philippe Boulet, Guy Brusselle, Roland Buhl, Lorenzo Cecchi, Denis Charpin, Elisio M Costa, Stefano Del Giacco, Marek Jutel, Ludger Klimek, Piotr Kuna, Daniel Laune, Mika Makela, Mario Morais-Almeida, Rachel Nadif, Marek Niedoszytko, Nikolaos G Papadopoulos, Alberto Papi, Oliver Pfaar, Daniela Rivero-Yeverino, Nicolas Roche, Boleslaw Samolinski, Mohamed H. Shamji, Aziz Sheikh, Charlotte Suppli Ulrik, Omar S Usmani, Arunas Valiulis, Arzu Yorgancioglu, Torsten Zuberbier, Joao A Fonseca, Benoit Pétré, Renaud Louis, Jean Bousquet

**Supplementary Methods**

**Study design**

In this cross-sectional study, we used MASK-air^®^ data to compare the frequency of occurrence (over the previous month) of five asthma symptoms in patients with probable asthma, possible asthma and no evidence of asthma.[E1] We assessed the frequency of dyspnea, wheezing, chest tightness, fatigue and night symptoms, as assessed using CARAT. We computed the sensitivity, specificity and predictive values of each individual symptom in differentiating (i) patients with probable or possible asthma versus no evidence of asthma and (ii) patients with probable versus no possible asthma (as determined by k-means cluster analysis).[E1] In addition, for patients with probable asthma, we assessed the control of asthma associated with the frequency of occurrence of each individual symptom. Results of this study were validated in a sample of patients in whom asthma diagnosis had been assessed by a physician in the context of a transfer of innovation project (Twinning) of the European Innovation Partnership on Active and Healthy Ageing.

**Settings and participants**

MASK-air^®^ has been launched in 27 countries (www.mask-air-com). It has been freely available in the Google Play and Apple App Stores since 2015. MASK-air^®^ is an mHealth app for digitally-enabled, patient-centred care in rhinitis and asthma multimorbidity as well as a Good Practice of OECD.[E2]

In this study, we included data from MASK-air^®^ users from May 21, 2015 to December 2021, reporting MASK-air^®^ data in at least three different months. The users (i) had a self-reported diagnosis of allergic rhinitis and (ii) ranged in age from 16 to 90 years (or lower than 16 years in countries with a lower age of digital consent).[E3;E4]

In addition, we included data from participants of the Twinning project, who were enrolled during a medical consultation with an asthma specialist in Germany, Italy, Poland or Portugal. [E1] Asthma was diagnosed according to the Global Initiative for Asthma (GINA),[E5] with patients having a pulmonary function test and answering to the CARAT questionnaire. Following that consultation, participants were classified as having “current asthma” or “no evidence of [current or past] asthma”.

**Ethics**

MASK-air^®^ has a CE1 marking. It follows the General Data Protection Regulation (GDPR).[E6] An Independent Review Board (Bohn-Köln) approval was obtained for the MASK-air studies.[E7] For the Twinning project, additional local review board approvals were obtained (Mannheim – reference: 2018-527N-MA, 29.03.2018 for Germany; Coimbra – reference: CHUC-022-18, 14.09.2018 for Portugal; Warsaw – reference: AKBE/213/2019, 13.05.2019 for Poland; Bari – reference: 7287, 30.03.2022 for Italy) and written consent was provided by patients. All data were anonymised before the study using k-anonymity and users agreed to the analysis of their data for research purposes in the terms of use (translated into all languages and customised according to the legislation of each country).

**Data sources and variables**

The MASK-air^®^ app comprises a daily monitoring questionnaire assessing (i) the impact of asthma and rhinitis symptoms on a daily basis by means of 0-100 visual analogue scales (VASs) (with a higher score corresponding to a higher impact of allergy symptoms) and (ii) the daily use of asthma and rhinitis medication (available from country-specific lists with prescribed and over-the-counter medications).[E3] The symptom and medication information provided in the MASK-air^®^ daily monitoring questionnaire allows for the computation of the e-DASTHMA, a 0-100 score assessing the daily control of asthma and which can be calculated by the following formula:[E8]

[(0.086 × VAS Asthma) + (1.756 if inhaled corticosteroid (ICS) without long-acting β2 agonist (LABA) are used) + (0.859 if ICS with LABA – excluding formoterol – are used) + (1.238 if ICS with formoterol is used) + (0.559 if short acting β2 agonist (SABA) or short acting muscarinic agent (SAMA) are used) + (4.022 if biologics or LAMA are used)] × 6.695

In addition to the MASK-air^®^ daily monitoring questionnaire, MASK-air^®^ also includes (although in a non-mandatory, non-daily basis) CARAT, a questionnaire assessing the control of allergic rhinitis and asthma in the previous four weeks (Table E1).[E9] CARAT is computed as the sum of the scores of its 10 symptoms (0-3), ranging from 0 to 30. It may be divided into two components: CARAT-rhinitis (questions 1-4; range=0-12) and CARAT-asthma (questions 5-10; range=0-18). A higher CARAT score indicates better control.

**Sample size**

Data from all users meeting the inclusion criteria were included. No sample size calculation was performed.

**Biases**

There are potential information biases related to the self-reported nature of the data collection. There may be an over-representation of users suffering from moderate-to-severe asthma[E10] and of younger individuals. We did not include users with asthma alone as their number is usually low[E11] and they represented less than 10% of the MASK-air^®^ database.

**Data analysis**

When responding to the MASK-air^®^ daily monitoring questionnaire, it is not possible to skip any of the questions and data are saved to the dataset only after the final answer. This precludes any missing data within each daily monitoring questionnaire. All analyses were performed using software R (version 4.0.0).

Using a two-step approach, k-means cluster analysis methods were applied to group MASK-air^®^ users on their probability of having asthma. The following variables were taken into account: (i) their self-reported asthma status (i.e., whether the patient self-reported to having asthma or not), (ii) the number of days using asthma medication and (iii) the maximum reported VAS asthma levels. Obtained clusters subsequently enabled the classification of patients as having “probable asthma”, “possible asthma” or “no evidence of asthma” (i.e., rhinitis alone), following a previously-described methodology.[E1]

For patients with “probable asthma”, “possible asthma” or “no evidence of asthma”, using the CARAT questions (Table E1), we assessed the frequency of having at least one day per week of (i) shortness of breath/dyspnea, (ii) wheezing in the chest, (iii) chest tightness, (iv) tiredness/limitations in doing tasks and (v) night symptoms. For each question, we calculated its sensitivity (probability of that symptom to have occurred at least once per week) in a patient with “possible” or “probable asthma”, its specificity (probability of that symptom not to have occurred in a patient with no “evidence of asthma”), its positive predictive value (PPV; probability of the patient having “possible” or “probable asthma” in the occurrence of the symptom) and its negative predictive value (NPV; probability of the patient having “no evidence of asthma” in the absence of the symptom). We also assessed the performance of each question in the discrimination between “possible asthma” and “probable asthma”. As each patient could have answered more than once to CARAT, we considered, in our main analysis, the first CARAT reported by each patient. We then performed a sensitivity analysis considering (i) all reported CARAT questionnaires or (ii) pre-COVID-19 data only.

To validate the obtained results, the sensitivity, specificity and predictive values of ever occurrence of each symptom in the discrimination of patients with “current asthma” *versus* “no evidence of asthma” were assessed in a sample of patients in which the diagnosis of asthma was established by a physician (Twinning participants).

Finally, in patients with probable asthma, we computed the median e-DASTHMA by each category of each CARAT question to assess the most discriminative symptom regarding asthma control. We considered (i) the median e-DASTHMA levels for the four weeks before answers to CARAT were provided (as CARAT assesses the period of four weeks) and (ii) the maximal e-DASTHMA level.

**References**

E1 - Bousquet J, Sousa-Pinto B, Anto J, Amaral R, Brussino L, et al. Identification by cluster analysis of patients with asthma and nasal symptoms using the MASK-air® mHealth app. Pulmonology. 2023;29(4):292-305.

E2 - Bousquet J, Bedbrook A, Czarlewski W, Onorato GL, Arnavielhe S, et al. Guidance to 2018 good practice: ARIA digitally-enabled, integrated, person-centred care for rhinitis and asthma. Clin Transl Allergy. 2019;9:16.

E3 - Bousquet J, Arnavielhe S, Bedbrook A, Bewick M, Laune D. MASK 2017: ARIA digitally-enabled, integrated, person-centred care for rhinitis and asthma multimorbidity using real-world-evidence. Clin Transl Allergy. 2018;8:45.

E4 - Bousquet J, Anto JM, Bachert C, Haahtela T, Zuberbier T, et al. ARIA digital anamorphosis: Digital transformation of health and care in airway diseases from research to practice. Allergy. 2021;76(1):168-90.

E5 - GINA report 2021. https://ginasthma.org/wp-content/uploads/2021/05/GINA-Main-Report-2021-V2-WMS.pdf

E6 - Laune D, Arnavielhe S, Viart F, Bedbrook A, Mercier J, et al. [Adaptation of the General Data Protection Regulation (GDPR) to a smartphone app for rhinitis and asthma (MASK-air(R))].Rev Mal Respir. 2019;36(9):1019-31.

E7 - Bousquet J, Agache I, Aliberti MR, Angles R, Annesi-Maesano I. Transfer of innovation on allergic rhinitis and asthma multimorbidity in the elderly (MACVIA-ARIA) - Reference Site Twinning (EIP on AHA). Allergy. 2018;73(1):77-92.

E8 - Sousa-Pinto B, Jacome C, Pereira AM, Regateiro FS, Almeida R, et al. Development and validation of an electronic daily control score for asthma (e-DASTHMA): a real-world direct patient data study. Lancet Digit Health. 2023;5(4):e227-e238.

E9 - Fonseca JA, Nogueira-Silva L, Morais-Almeida M, Azevedo L, Sa-Sousa A, et al. Validation of a questionnaire (CARAT10) to assess rhinitis and asthma in patients with asthma. Allergy. 2010;65(8):1042-8.

E10 - Bedard A, Basagana X, Anto JM, Garcia-Aymerich J, Devillier P, et al. Treatment of allergic rhinitis during and outside the pollen season using mobile technology. A MASK study. Clin Transl Allergy. 2020;10(1):62

E11 - Leynaert B, Neukirch C, Liard R, Bousquet J, Neukirch F. Quality of life in allergic rhinitis and asthma. A population-based study of young adults. Am J Respir Crit Care Med. 200; 162(4 Pt 1):1391-6.

**Table E1: CARAT questionnaire – the CARAT questionnaire is computed as the sum of the score of its 10 symptoms**

| **Question** | **Answer options** |
| --- | --- |
| **During the last 4 weeks, because of your asthma/rhinitis/allergy, how many times, on average, did you experience:** | |
| 1. Blocked nose? | Never  Up to 2 days per week  More than 2 days per week  Almost every day or every day |
| 2. Sneezing? |  |
| 3. Itchy nose? |  |
| 4. Runny nose? |  |
| 5. Shortness of breath/dyspnoea? |  |
| 6. Wheezing in the chest? |  |
| 7. Chest tightness upon physical exercise? |  |
| 8. Tiredness/limitations in doing daily tasks? |  |
| 9. Waking up during the night because of your asthma/rhinitis/allergy? |  |
| **During the last 4 weeks, because of your asthma/rhinitis/allergy, how many times did you have to:** | |
| 10. Increase the use of your medications? | I am not taking any medicines  Never  Less than 7 days  7 or more days |

**Table E2: Characteristics of days when answers to CARAT were provided**

|  | **First CARAT assessment per user**  **(*N*=951)** | **All CARAT assessments**  **(*N*=2154)** |
| --- | --- | --- |
| CARAT – median (IQR) | 18 (9) | 16 (11) |
| Controlled – *N* (%) | 119 (12.5) | 302 (14.0) |
| CARAT-rhinitis – median (IQR) | 5 (6) | 4 (7) |
| CARAT-asthma – median (IQR) | 13 (5) | 11 (7) |
| Ever occurrence of symptoms in the previous month – *N* (%) |  |  |
| Shortness of breath/dyspnea | 515 (54.2) | 1419 (65.9) |
| Wheezing in the chest | 327 (34.4) | 893 (41.5) |
| Chest tightness | 354 (37.2) | 1066 (49.9) |
| Tiredness/limitations doing tasks | 568 (59.7) | 1427 (66.2) |
| Night symptoms | 524 (55.1) | 1201 (55.8) |
| VAS asthma – median (IQR) | 4 (17) | 8 (45) |
| eDASTHMA – median (IQR) | 2.9 (14.8) | 8.1 (29.8) |
| Days reporting asthma medication – *N* (%) |  |  |
| ICS alone | 51 (5.4) | 267 (12.4) |
| ICS+Formoterol | 65 (6.8) | 172 (8.0) |
| ICS+LABA (except formoterol) | 51 (5.4) | 263 (12.2) |
| SABA or SAMA | 34 (3.6) | 189 (8.8) |
| Biological drugs or LAMA | 14 (1.5) | 36 (1.7) |

CARAT=Control of Allergic Rhinitis and Asthma Test; ICS=Inhaled corticosteroids; IQR=Interquartile range; LABA=Long-acting β-agonists; LAMA=Long-acting muscarinic antagonists; SABA=Short-acting β-agonists; SAMA=Short-acting muscarinic antagonists
